# Integrating Metabolic and Gene Expression Profiling of Glucosinolate Biosynthesis Under Drought Stress in *Brassica oleracea*

**DOI:** 10.3390/ijms27031598

**Published:** 2026-02-06

**Authors:** Hajer Ben Ammar, Souhir Kabtni, Donata Arena, Marwen Amari, Nicolas Al Achkar, Ferdinando Branca, Sonia Marghali

**Affiliations:** 1 Crop Science Department, Agricultural Institute of Slovenia, 1000 Ljubljana, Slovenia; 2 Laboratory of Microorganisms and Active Biomolecules (LR03ES03), Department of Biology, Faculty of Sciences of Tunis, Tunis El-Manar University, University Campus, Tunis 2092, Tunisia; souhir.kabtni@fst.utm.tn (S.K.); sonia.marghali@fst.utm.tn (S.M.); 3 Department of Agriculture, Food and Environment (Di3A), University of Catania, Via Valdisavoia 5, 95123 Catania, Italy; donata.arena@unict.it (D.A.); nicolas.alachkar@phd.unict.it (N.A.A.); fbranca@unict.it (F.B.); 4 Department of Earth and Marine Sciences (DiSTeM), University of Palermo, Via Archirafi 22, 90123 Palermo, Italy; marwenameri91@gmail.com

**Keywords:** *Brassica oleracea* L., glucosinolates, gene expression, qRT-PCR, abiotic stress response

## Abstract

Drought stress induces pronounced metabolic and transcriptional reprogramming of glucosinolate (GLS) biosynthesis in *Brassica oleracea*. An integrative approach combining HPLC-based quantification of individual GLSs, quantitative real-time PCR of core biosynthetic and regulatory genes, correlation-based network analysis, and in silico promoter characterization was applied to evaluate drought responses across genetically diverse accessions. Drought triggered strong, accession-specific shifts in GLS composition, with sinigrin content increasing from 35.9% to 55.1% in BR1 and glucoerucin reaching up to 80.2% in CCP1, while indolic GLSs such as glucobrassicin and neoglucobrassicin accounted for >75% of total GLSs in CV2 and CCP3. Hierarchical clustering separated accessions into four distinct drought response clusters independent of morphotype. Correlation analysis revealed drought-induced rewiring of GLS interdependencies, characterized by strengthened positive associations among aliphatic GLSs (r > 0.75). Gene expression profiling identified conserved MYB-centered regulatory modules (*MYB28*, *MYB29*, *MYB34*, *MYB122*) alongside strong accession-specific induction of *CYP79F1* (up to 6.3-fold), *FMOGS-OX5* (up to 4.8-fold), and *ST5a* (up to 5.1-fold). Promoter analysis revealed enrichment of ABA- and stress-responsive cis-regulatory elements. These findings delineate a genotype-dependent regulatory framework underlying GLS plasticity and identify quantitative metabolic and transcriptional markers relevant for breeding drought-resilient *Brassica* cultivars.

## 1. Introduction

Drought stress represents one of the most severe constraints on plant productivity, affecting not only growth and yield but also crop quality and adaptive fitness [1]. In response to water limitation, plants reprogram nutrient allocation, reorganize metabolic networks, and redistribute metabolites to maintain cellular homeostasis and survival. These adjustments are particularly evident in secondary metabolism, where trade-offs between growth, defense, and stress tolerance are tightly regulated [2].

The genus *Brassica* represents one of the most biologically and agronomically significant groups of crop plants, comprising vegetables, oilseed crops, and condiment species that collectively contribute substantially to global food and nutritional security. Owing to its well-defined polyploid evolutionary history, extensive interspecific diversity, and exceptional capacity for secondary metabolite diversification, *Brassica* has become a reference system for studying the genetic and environmental regulation of specialized metabolism. Within this framework, *Brassica* species provide a powerful model for investigating drought-induced regulation of secondary metabolism due to their characteristic glucosinolate (GLS)-rich profiles. *Brassica oleracea* L., which includes broccoli, cabbage, kale, and cauliflower, is both economically and nutritionally important and displays important morphological and genetic diversity [3]. Traditional landraces constitute a key reservoir of adaptive and biochemical variation, offering valuable resources for breeding resilient and nutritionally improved cultivars. Recent studies have documented extensive diversity among *B. oleracea* landraces at both the genetic and GLS compositional levels, reinforcing their importance for crop improvement and quality traits [4,5,6]. From a dietary perspective, *Brassica* vegetables are major sources of GLSs and their bioactive hydrolysis products, sustaining strong interest in strategies to optimize GLS composition in crops [7,8].

Glucosinolates (GLSs) play central roles in the ecological and physiological performance of *Brassica* species by mediating defense against herbivores and pathogens [9]. In humans, GLS-derived isothiocyanates and related compounds are implicated in cancer prevention, detoxification, and anti-inflammatory processes [10]. Importantly, *Brassica* crops exhibit extraordinary quantitative and qualitative variation in GLS accumulation, not only between species but also among cultivars of the same species [11,12], reflecting the strong genetic and environmental control of this pathway. Environmental factors including light [13,14], temperature [15,16], salinity [17], elicitors [18], and drought stress [10] markedly reshape GLS profiles, indicating that glucosinolate metabolism constitutes a highly plastic interface between plant stress physiology and nutritional quality. Structurally, GLSs are β-thioglucosides-N-hydroxysulfates derived from amino acids and are classified into aliphatic, indolic, and aromatic groups [3]. Aliphatic GLSs, derived mainly from methionine, dominate in vegetables such as broccoli, cabbage, and kale, whereas indolic GLSs originate from tryptophan and are enriched in crops such as Brussels sprouts and cauliflower [17]. Aromatic GLSs, derived from phenylalanine and tyrosine, characterize species such as watercress and rocket [3]. Biochemical diversification has major implications for sensory attributes and the spectrum of health-promoting phytochemicals and the breakdown products in plant-derived matrices [19].

Under drought stress, plants reorganize resource allocation to prioritize essential physiological functions. This often involves restricting nitrogen, sulfur, and carbon fluxes toward growth and primary metabolism, thereby constraining the synthesis of nitrogen-rich secondary metabolites such as GLSs [20]. These adaptive shifts are mediated by complex regulatory networks that integrate transcriptional control with epigenetic regulation, metabolic feedback and hormonal signaling [21]. As a result, drought does not uniformly reduce GLS levels but instead reshapes GLS profiles in a compound- and genotype-dependent manner, favoring metabolites that enhance stress tolerance while suppressing others. From an applied perspective, understanding these stress-associated shifts is crucial for maintaining nutritional quality while improving drought resilience in *Brassica* crops [22,23]. At the molecular level, GLS biosynthesis proceeds through amino acid side-chain elongation, core structure formation, and side-chain modification [24]. This pathway has been extensively elucidated in *Arabidopsis thaliana*, leading to the identification of key biosynthetic genes [25]. The MAM1–3 genes control side-chain elongation, a critical determinant of aliphatic GLS diversity [26,27], while MYB transcription factors such as MYB28, MYB29, and MYB34 act as master regulators of GLS biosynthetic gene expression [28]. Comparative genomics has confirmed both the conservation and diversification of these regulatory modules across Brassicaceae, providing robust reference frameworks for crop-level studies [29,30].

Despite extensive characterization of glucosinolate biosynthetic genes and their transcriptional regulators, a critical gap remains: transcript abundance frequently fails to accurately predict glucosinolate accumulation, particularly under environmentally induced stress conditions [24]. This uncoupling reflects the intrinsically multilayered regulation of secondary metabolism, in which transcriptional programs are further shaped by post-transcriptional control, enzyme turnover and stability, precursor availability, metabolic feedback regulation, and phytohormone-dependent signaling networks [31]. Under water deficit, additional constraints on nitrogen, sulfur, and carbon metabolism further reshape metabolic flux through the GLS pathway, frequently overriding transcriptional signals and generating genotype-specific metabolic outcomes. Consequently, genetic variation in regulatory architecture and metabolic buffering capacity is expected to determine how individual genotypes translate transcriptional responses into glucosinolate phenotypes under drought [32]. Within this framework, the present study examines intraspecific variation in GLS composition across twelve genetically diverse *Brassica oleracea* landraces and a composite cross population grown under contrasting water regimes. By integrating glucosinolate profiling with expression analysis of key genes controlling side-chain elongation, core biosynthesis, and transcriptional regulation, we aim to resolve how genotype × drought interactions shape the relationship between gene expression and metabolite accumulation. This integrative approach provides a mechanistic basis for identifying drought-resilient, nutritionally optimized Brassica genotypes and for exploiting natural regulatory variation in breeding programs.

## 2. Results

### 2.1. Drought-Induced Variability in Glucosinolate Accumulation

**Figure 1:**
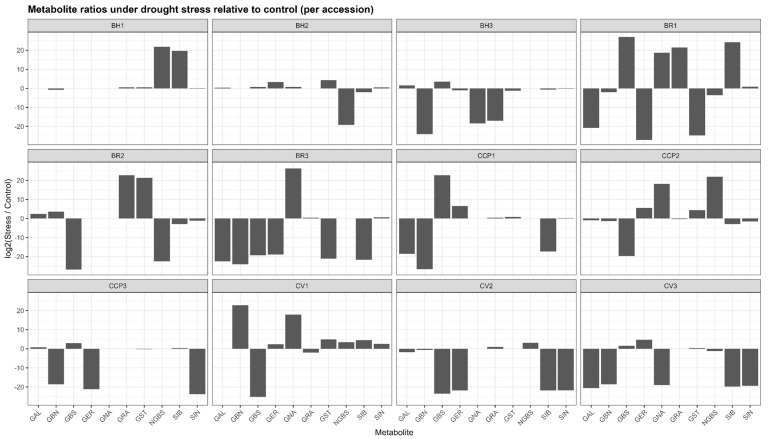
Accession-dependent shifts in glucosinolate ratios under drought stress. GAL: glucoalyssin, GBN: glucobrassicanapin, GBS: glucobrassicin, GER: glucoerucin, GNA: gluconapin, GRA: glucoraphanin, GST: gluconasturtiin, NGBS: neoglucobrassicin, SIB: sinalbin, SIN: sinigrin.

Drought stress induced pronounced yet highly heterogeneous changes in glucosinolate (GLS) profiles across accessions, as revealed by log_2_(stress/control) ratios (Figure 1). Rather than coordinated shifts across all metabolites, responses were dominated by compound-specific and accession-specific polarization, with log_2_ values ranging from extreme negative to extreme positive within the same genotype. Across the dataset, log_2_ values spanned from near zero, indicating biochemical stability, to extreme positive or negative values exceeding ±20, reflecting qualitative metabolite switching rather than proportional variation. In BH1, most GLSs exhibited minimal log_2_ changes (e.g., sinigrin (SIN) and glucoerucin (GER) close to 0), whereas neoglucobrassicin (NGBS) and sinalbin (SIB) showed very strong positive log_2_ responses (>+19), consistent with de novo induction under stress. BH2 displayed broader activation, with strong positive log_2_ shifts for gluconasturtiin (GST) (+4.3) and glucoerucin (GER) (+3.3), while neoglucobrassicin was completely suppressed (log_2_ < −19). In BH3, drought triggered a marked redistribution characterized by induction of glucobrassicin (GBS) (+3.5) and glucoalyssin (GAL) (+1.6), concomitant with strong negative log_2_ responses for several aliphatic GLSs, including glucobrassicanapin (GBN) (log_2_ < −24).

The BR accessions exhibited the most extreme reconfiguration. BR3 showed a dominant positive log_2_ response for gluconapin (GNA≈ +26), while nearly all other GLSs were strongly repressed (log_2_ < −18), indicating near-exclusive metabolic channeling. BR1 and BR2 similarly displayed highly polarized profiles, combining strong induction (log_2_ > +20) of selected aliphatic and/or aromatic GLSs (GRA, GST, and SIB) with complete repression of others (log_2_ < −25). Among the CCP lines, CCP1 and CCP2 showed exceptionally strong induction of glucoerucin (GER ≈ +6.6 and +5.6, respectively) and moderate induction of gluconasturtiin (GST) (+4–4.5), whereas CCP3 exhibited suppression of most aliphatic GLSs (GER and SIN) with selective induction of glucobrassicin (GBS) (+2.9). Cauliflower accessions also displayed selective responses, with CV1 showing strong positive log_2_ shifts for gluconasturtiin (GST) (+4.9) and glucobrassicanapin (GBN) (+22), and CV2 characterized by a pronounced induction of neoglucobrassicin (NGBS) (+3.0) alongside broad suppression of other GLSs. Overall, drought stress reshaped GLS profiles through selective, quantitative, and switch-like biochemical responses, underscoring substantial metabolic plasticity among accessions.

Several accessions (e.g., BH1, BH2) exhibited relatively narrow log_2_ distributions, indicating biochemical stability, whereas others showed marked profile restructuring driven by qualitative metabolite switching. In BR3, drought response was almost entirely dominated by a strong positive log_2_ shift of gluconapin (GNA), while most other GLSs displayed large negative log_2_ values, reflecting near-exclusive metabolic channeling. Similarly, BR1 and BR2 showed highly polarized responses characterized by strong induction of selected aliphatic or aromatic GLSs alongside complete repression of others. In the CCP lines, drought triggered contrasting patterns: CCP1 and CCP2 exhibited strong positive log_2_ shifts in glucoerucin and gluconasturtiin, whereas CCP3 displayed suppression of aliphatic GLSs with concurrent induction of glucobrassicin (GBS). Among cauliflower accessions, CV1 showed pronounced selectivity, with strong positive log_2_ responses limited to GBN and GNA and widespread repression of the remaining GLSs. The aliphatic GLSs displayed the widest log_2_ amplitude, while indolic (GBS and NGBS) and aromatic (GST and SIB) GLSs frequently contributed to the strongest positive responses in specific genotypes.

**Figure 2:**
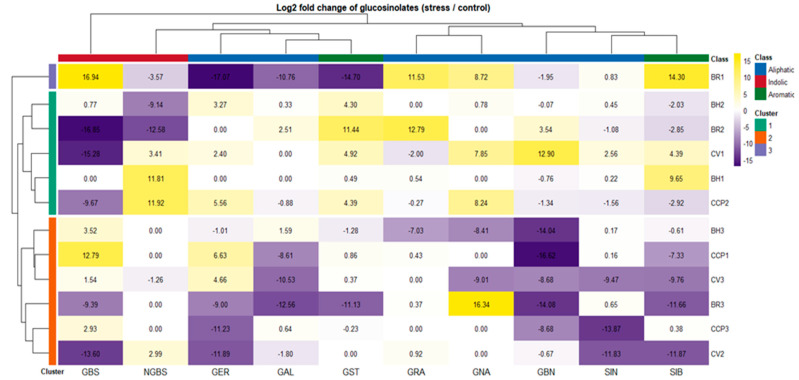
Differential glucosinolate responses to drought stress across Brassica accessions. Heatmap shows the log_2_ fold change (log_2_FC) of individual glucosinolates in drought-stressed plants relative to control conditions. Positive log_2_FC values (yellow) indicate increased glucosinolate accumulation under stress, whereas negative values (purple) indicate reduced levels. Rows correspond to individual accessions and are hierarchically clustered, revealing distinct accession-specific glucosinolate response patterns to drought. Columns represent individual glucosinolates clustered according to similarities in their stress-induced behavior. Colored row annotations indicate accession cluster membership, while column annotations denote glucosinolate biochemical classes (aliphatic, indolic, and aromatic). Cell labels represent the exact log_2_ fold change values calculated from the raw data reported in Table S1.

Drought-induced changes in glucosinolate (GLS) composition were assessed using log_2_ fold-change values relative to control conditions using absolute concentrations reported in Table S1. These fold-change values are summarized as a hierarchically clustered heatmap in Figure 2, enabling direct visualization of accession-dependent reprogramming across aliphatic (GRA, GNA, GER, GAL, GBN, SIN), indolic (GBS, NGBS) and aromatic (GST, SIB) GLS classes. Overall, drought triggered highly heterogeneous GLS adjustments with clustering patterns not reflecting morphotype or species grouping, indicating that GLS plasticity under water limitation is largely controlled at the accession level rather than by broad taxonomic identity. Hierarchical clustering separated accessions into three major response groups with contrasting metabolic behaviors. Cluster 1 (BH2, BR2, CV1, BH1, and CCP2) exhibited the most pronounced but variable drought responsiveness, characterized by strong induction of aromatic and aliphatic GLSs depending on genotype. This group was dominated by the stimulation of GST, which reached particularly high induction in BR2, while BH1 exhibited only a minor aromatic glucosinolate GST induction compared with the other accessions. Notably, within Cluster 1, accessions diverged in their indolic modulation: BH2 and BR2 were associated with repression of NGBS, whereas CV1, BH1, and CCP2 displayed increased NGBS, suggesting distinct regulatory control points within the indolic branch.

In contrast, BR1 formed an isolated Cluster 2, reflecting a unique and polarized drought signature. This accession combined strong induction of GBS, GRA, GNA, and SIB, with simultaneous repression of several other GLSs including GER, GAL, GST, and NGBS, indicating a selective redirection of GLS flux rather than a generalized pathway activation. Finally, Cluster 3 (BH3, CCP1, CV3, BR3, CCP3, and CV2) exhibited an overall attenuated drought response, marked by negative fold-changes for several GLSs and, in particular, limited responsiveness of GBN across genotypes. Within this cluster, two coherent response modules could be resolved. BH3, CCP1, and CV3 consistently exhibited GBS induction, indicative of a preferential shift toward the indolic branch, coupled with SIB downregulation. In contrast, BR3, CCP3, and CV2 shared a similar overall drought signature, showing GER repression, while maintaining near-stable GRA levels, suggesting a more conservative adjustment of aliphatic GLS biosynthesis.

These patterns suggest two predominant biochemical strategies under drought: one characterized by the accumulation of indolic/aromatic GLSs (GBS, NGBS, GST), and the other dominated by aliphatic GLSs (GER). Figure 2 demonstrates that drought induces distinct, accession-specific GLS adjustment patterns, ranging from strong metabolic reprogramming to repression-dominant stability, rather than uniform pathway-level responses.

### 2.2. Correlation Shifts Among Glucosinolates in Response to Drought Stress

**Figure 3:**
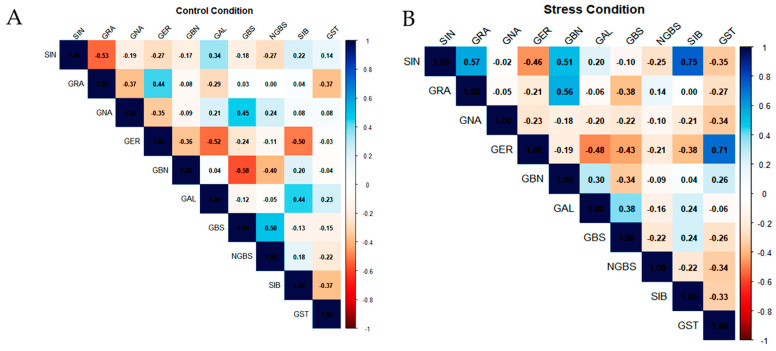
Pearson correlation between different glucosinolate contents in leaves under (**A**) control conditions and (**B**) drought stress conditions. Colors represent the strength and direction of correlations. Correlation strength was interpreted based on r values (weak: |r| < 0.3; moderate: 0.3 ≤ |r| < 0.6; strong: |r| ≥ 0.6).

The Pearson correlation matrices (Figure 3A,B) describe the pairwise relationships among the quantified glucosinolates (GLSs) in *Brassica oleracea* genotypes under control and drought stress conditions. Under control conditions (Figure 3A), both negative and non-significant correlations were observed. A moderate negative correlation was recorded between sinigrin (SIN) and glucoraphanin (GRA) (r = −0.53), as well as between glucoerucin (GER) and glucobrassicanapin (GBN) (r = −0.36). Additionally, GBN was negatively correlated with glucobrassicin (GBS) (r = −0.58). In contrast, certain GLS pairs, such as GRA and neoglucobrassicin (NGBS), exhibited no correlation.

Under drought stress conditions (Figure 3B), the glucosinolate correlation network was markedly reconfigured, indicating a profound reorganization of pathway coordination. Sinigrin (SIN) became positively associated with both glucoraphanin (GRA; r = +0.57) and sinalbin (SIB; r = +0.75). In parallel, glucoerucin (GER) showed a strong positive correlation with gluconasturtiin (GST; r = +0.71), while displaying negative associations with glucobrassicin (GBS; r = −0.43) and glucoalyssin (GAL; r = −0.48), pointing to a drought-induced shift in metabolic flux away from indolic and specific aliphatic derivatives. A weak negative correlation was also observed between GBS and neoglucobrassicin (NGBS; r = −0.22), indicating partial uncoupling within the indolic glucosinolate subgroup. Notably, the overall density of moderate to strong correlations increased under drought stress, reflecting a tighter coordination of glucosinolate metabolism in response to limited water availability.

### 2.3. Expression of Genes Involved in Glucosinolate Biosynthesis

**Figure 4:**
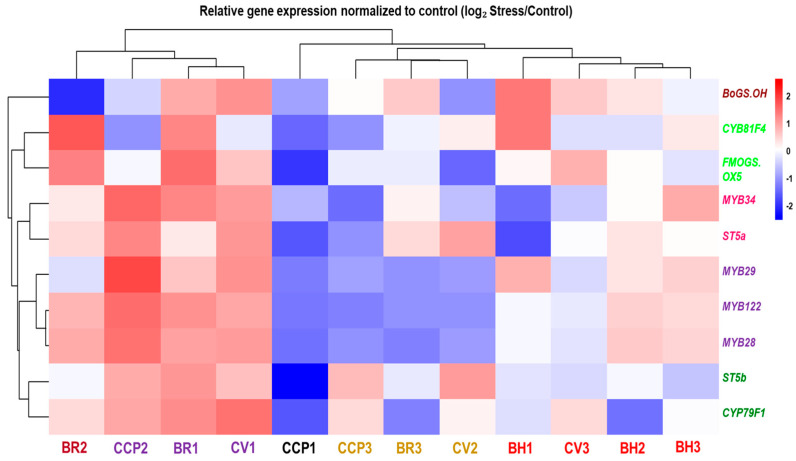
Hierarchical clustering and heatmap of gene expression profiles involved in glucosinolate biosynthesis across *Brassica oleracea* accessions under drought stress. Expression levels (log2-transformed) are shown for transcription factors (*MYB28*, *MYB29*, *MYB34*), core biosynthetic enzymes (*CYP79F1*, *FMOGS-OX5*, *ST5a*), and the late-pathway gene *BoGS_OH*. Accessions and genes belonging to the same cluster are represented by the same color in the dendrogram.

Hierarchical clustering of drought-responsive expression profiles (log_2_ Stress/Control) revealed pronounced genotype-dependent transcriptional reprogramming of glucosinolate (GLS)-related genes across the evaluated Brassica accessions (Figure 4; Table S2). At the accession level, samples segregated into distinct response modules reflecting differences in both response magnitude and direction. A first transcriptional module, comprising CCP2, BR1, and CV1, was characterized by coordinated induction of multiple GLS-related transcripts, including the biosynthetic/modification genes FMOGS_OX5, ST5a, and ST5b, together with MYB regulators (MYB28/MYB29 and MYB34/MYB122), indicating strong co-regulation across regulatory and enzymatic components of the pathway. Notably, within this module, CCP2 displayed a clear deviation at the tailoring level, with CYP81F4 and BoGS_OH showing downregulation despite the broad induction trend of most other targets. Closely associated with this module, BR2 exhibited a mixed-direction profile, with induction of several transcripts (including FMOGS_OX5 and CYP81F4) but strong repression of BoGS_OH, confirming that drought responses are not uniformly directional within a single genotype. A second response module, represented by CCP3, BR3, and CV2, showed predominantly negative regulation of MYB transcription factors combined with gene-selective regulation among structural and modification genes, consistent with partial uncoupling between regulatory and downstream pathway components. In contrast, CCP1 exhibited the most extreme repression-dominant pattern, characterized by pronounced downregulation across nearly all targets, including strong repression of ST5b and FMOGS_OX5 together with broad attenuation of MYB expression. Within the BH group, responses were heterogeneous: BH1 displayed strong induction of BoGS_OH and CYP81F4 while several MYB regulators were weakly responsive or downregulated, whereas BH2 and BH3 showed comparatively attenuated transcriptional shifts, including only mild induction of selected genes (e.g., MYB34 in BH3) and near-neutral regulation for several other transcripts. Collectively, these data indicate that drought modulates GLS-related transcription through multiple accession-dependent configurations, spanning coordinated co-induction, repression-dominant regulation, and selective uncoupling between upstream regulators and downstream biosynthetic/tailoring genes.

### 2.4. Correlation Between the Genes and the Profile of Glucosinolate

**Figure 5:**
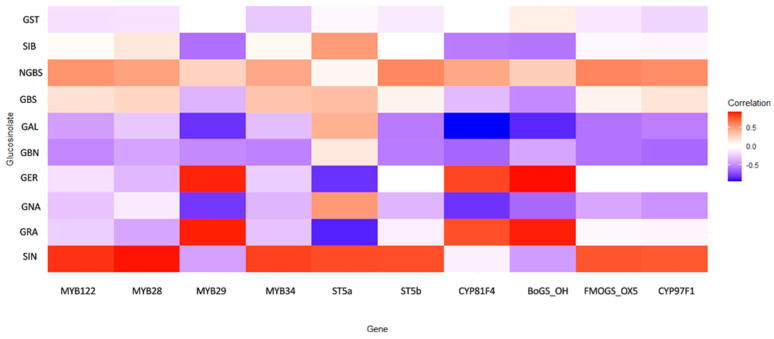
Heatmap of gene expression patterns and their association with glucosinolate biosynthetic profiles across *Brassica oleracea* accessions under drought stress. Colors represent the strength and direction of correlations. Correlation strength was interpreted based on r values (weak: |r| < 0.3; moderate: 0.3 ≤ |r| < 0.6; strong: |r| ≥ 0.6).

Through integrative correlation analysis of key transcription factors, biosynthetic genes, and metabolite profiles, we uncovered a structured regulatory architecture that delineates functional modules within the GLS metabolic network (Figure 5).

The MYB transcription factors emerged as central regulators of the aliphatic GLS pathway. *MYB28* and *MYB122* showed strong positive correlations with sinigrin (SIN) (r = 0.900 and 0.845, respectively), confirming their role as primary transcriptional activators of aliphatic GLS biosynthesis. *MYB29* displayed a dualistic behavior: a moderate negative correlation with SIN (r = −0.409) and a strong positive correlation with glucoraphanin (GRA) (r = 0.883), suggesting it may redirect metabolic flux between different aliphatic compounds. *MYB28* and *MYB29* also correlated with *CYP79F1*, a key enzyme catalyzing early steps in the aliphatic pathway. In contrast, *MYB34* showed weak or negative correlations (−0.2 < r < −0.3) with aliphatic GLSs, but moderate positive correlations (0.2 < r < 0.4) with indolic glucosinolates, including glucobrassicin (GBS) and neoglucobrassicin (NGBS). This expression pattern confirms its functional specialization for indolic GLS regulation. *ST5a* and *ST5b*, putative sulfotransferases involved in terminal GLS assembly, showed strong positive correlations with SIN (r = 0.782 and 0.777, respectively). Notably, *ST5a* exhibited a strong negative correlation with GRA (r = −0.841), indicating selective substrate routing favoring SIN accumulation. These findings suggest that final enzymatic steps actively contribute to shaping the GLS profile rather than operating constitutively. Genes involved in side-chain modification, particularly *CYP81F4* and *BoGS_OH*, exhibited strong negative correlations with gluconapin (GNA) (r = −0.791 and −0.610, respectively), and positive associations with GER and GRA. This divergent behavior indicates a regulatory branching point at the level of hydroxylation and methoxylation, potentially governed by *CYP81F4* activity. *FMOGS-OX5* and *CYP97F1*, which participate in secondary modifications, showed only weak and diffuse correlations. *BoGS_OH*, a gene of currently uncharacterized function within GLS biosynthesis, displayed consistently strong positive correlations with key aliphatic GLSs, including SIN and GBN (r > 0.7). Its transcriptional behavior implies a potential role in biosynthesis or transport, meriting further functional validation. Similarly, *ST5b* exhibited correlations with both aliphatic and indolic GLSs, suggesting dual-pathway involvement, possibly in terminal sulfation of diverse GLS cores.

### 2.5. Promoter Region Analysis for Cis-Acting Elements

**Figure 6:**
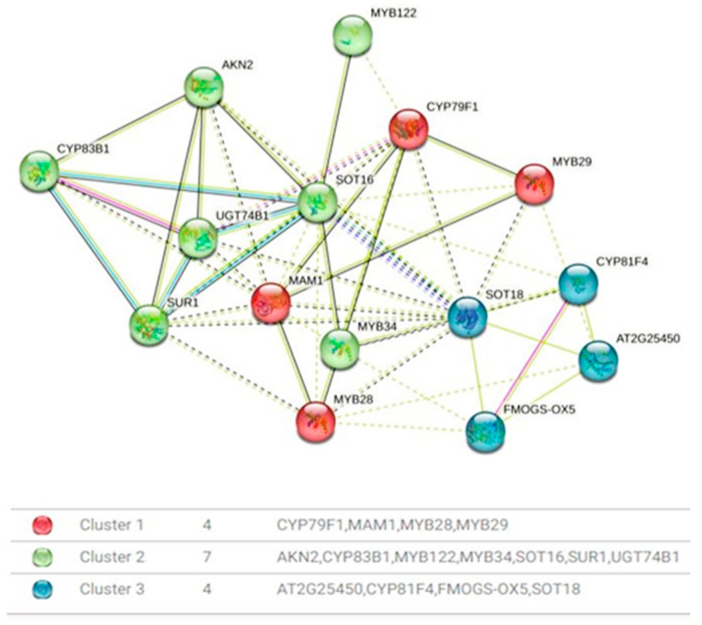
Protein–protein interaction network. Cluster 1 is shown in red: CYP79F1, MAM1, MYB28, and MYB29. Cluster 2 is shown in green: AKN2, CYP83B1, MYB122, MYB34, SOT16, SUR1, and UGT74B1. Cluster 3 is shown in blue: AT2G25450, CYP81F4, FMOGS-OX5, and SOT18. Solid lines indicate high-confidence interaction, while dashed lines represent predicted association. Edge Colors represent evidence sources (experimental, database, co-expression, etc.).

The presence of distinct protein clusters suggests an integrated response to various cues, ultimately fine-tuning the production of GLSs. This precision is paramount for the plant’s defense mechanisms against herbivores and pathogens, and it profoundly influences the flavor profile and nutritional value of cruciferous vegetables. In the protein–protein interaction network analysis, distinct clusters of proteins closely associated with the regulation of genes relevant to GLSs biosynthesis have been identified (Figure 6). Cluster 1, designated as the aliphatic glucosinolate biosynthesis regulation cluster, is distinguished by the presence of *CYP79F1*, a versatile enzyme responsible for catalyzing the conversion of short-chain elongated methionine into specific aldoximes, namely 5-methylthiopentanaldoxime, 6-methylthiohexanaldoxime, and 7-methylheptanaldoxime. Additionally, this cluster encompasses MYB28 and MYB29, transcription factors recognized as major regulators of aliphatic glucosinolate biosynthesis. The proximity of *CYP79F1* to *MYB28* and *MYB29* suggests a potential involvement in the glucosinolate biosynthesis pathway. Cluster 2 highlights a functional module comprising *SOT18*, *FMOGS-OX5*, *CYP81F4*, and *AT2G25450*. *SOT18*, an aliphatic desulfoglucosinolate sulfotransferase, plays a crucial role in the sulfate conjugation of desulfo-glucosinolates, particularly long-chain varieties. *FMOGS-OX5*, a flavin-monooxygenase glucosinolate S-oxygenase, is tasked with converting methylthioalkyl glucosinolates into methylsulfinylalkyl glucosinolates, thereby enriching glucosinolate diversity. *CYP81F4* is involved in indolic glucosinolate biosynthesis, catalyzing hydroxylation reactions. Meanwhile, *AT2G25450* contributes to the hydroxylation of but-3-enyl glucosinolates, yielding the toxic 2-hydroxybut-3-enyl glucosinolates. Cluster 3, denoted as Transcriptional Regulators, consolidates *MYB122*, *MYB34*, and *MYB29*, all serving as transcription factors with notable impacts on glucosinolate biosynthesis. *MYB122*, belonging to the *R2R3* factor gene family, is presumed to exert transcriptional control over the network. *MYB34* plays a pivotal role in modulating the expression of ASA1, a crucial control point in the tryptophan pathway. Although *MYB29*’s role in aliphatic glucosinolate biosynthesis is minor, it holds significance in promoting glucosinolate production and deterring insect herbivores.

**Figure 7:**
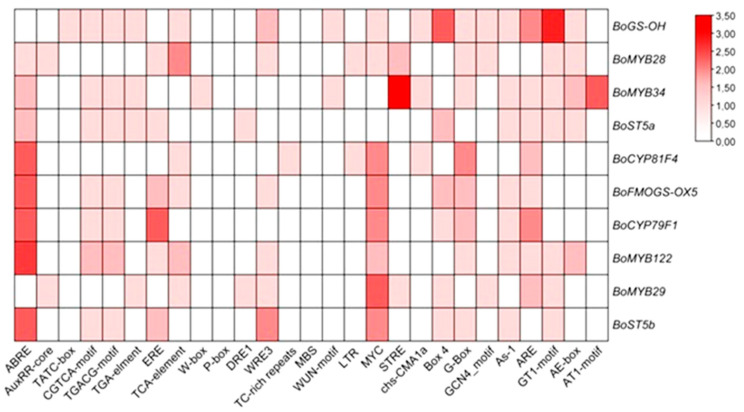
The cis-acting elements (CAEs) were detected by the online tool PlantCARE.

An in silico analysis of promoter regions from glucosinolate (GLS) biosynthetic and regulatory genes was undertaken to characterize the cis-regulatory landscape underlying GLS pathway control, with a focus on cis-acting elements linked to phytohormone signaling, abiotic stress responses, and developmental regulation (Figure 7).

The promoter analysis revealed a strong enrichment of CAEs linked to abiotic stress-responsive pathways, highlighting the potential integration of environmental cues into the transcriptional control of GLS metabolism. Notably, several hormone-responsive elements were identified, including ABRE (abscisic acid), CGTCA-motif (jasmonic acid), ERE (ethylene), GARE-motif (gibberellins), and the TCA-element (salicylic acid), suggesting a multilayered regulatory framework mediated by phytohormonal cross-talk. Among these, the ABRE and MYC motifs, associated with ABA signaling and stress-related transcription factors, respectively, were the most prevalent across the promoters analyzed, underscoring the likely central role of ABA-dependent signaling cascades in modulating GLS gene expression under abiotic stress conditions.

Additionally, several elements implicated in growth and developmental processes were detected, including the G-box (a binding site for bZIP and bHLH transcription factors) and the ARE element, which is responsive to hypoxic or anaerobic conditions. These findings collectively support the hypothesis that GLS biosynthesis is subject to complex transcriptional regulation involving both hormonal signaling and stress-responsive networks, thereby enabling plants to fine-tune their secondary metabolism in accordance with developmental cues and environmental challenges.

**Figure 8:**
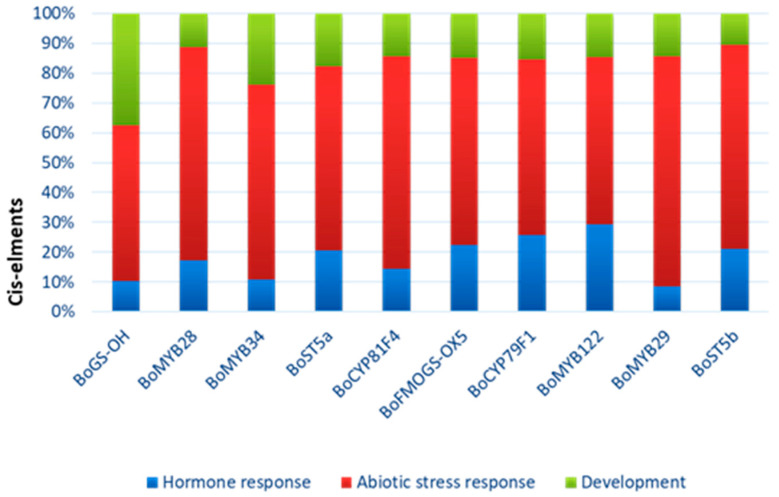
Predicted functions of the different CAEs present in promoter regions of Glucosinolate genes. The functions are categorized into three main biological processes: hormone response (blue), abiotic stress response (red) and development (green).

To further characterize the potential biological roles of glucosinolate-related genes in *Brassica oleracea*, we performed a functional categorization based on predicted involvement in hormone signaling, abiotic stress responses, and developmental processes derived from bioinformatic annotation and published literature (Figure 8).

Most genes, including *BoMYB28*, *BoMYB29*, *BoMYB34*, *BoST5a*, *BoCYP79F1*, and *BoFMOGS-OX5*, exhibit predominant putative functional annotation related to abiotic stress response. Several genes, particularly transcription factors, also show significant involvement in hormone-related pathways. *BoGS-OH* shows a more balanced functional distribution, including a notable proportion associated with developmental processes. This functional classification highlights the multi-layered regulatory roles of glucosinolate pathway genes in stress adaptation and plant development.

## 3. Discussion

### 3.1. Genotype-Specific Glucosinolate Plasticity and Qualitative Metabolic Switching Under Drought

The drought-induced glucosinolate (GLS) responses observed in this study highlight pronounced accession-specific metabolic reprogramming rather than a conserved, species-level pattern, a phenomenon that is increasingly recognized in Brassicaceae stress physiology [3,33]. Across the twelve *Brassica* accessions examined, drought responses ranged from relative metabolic stability (e.g., BH1) to extreme compound-specific induction or repression (e.g., BR3, CV2), illustrating the intrinsic plasticity of GLS metabolism under abiotic stress. Hierarchical clustering based on fold-change profiles further revealed that drought-responsive GLS modulation did not align with morphotype or species classification but instead reflected distinct biochemical response modes among accessions. Similar genotype-dependent variability has been reported in previous studies examining drought effects on GLS accumulation, where both increases and decreases in total and individual GLSs were observed depending on genetic background, stress severity, and experimental conditions [3,17,34,35,36]. Notably, several of the most pronounced responses were driven by qualitative metabolic switches, whereby compounds absent or present only at trace levels under control conditions became dominant under drought stress. These de novo accumulation events, reflected by very high log_2_ stress/control ratios, indicate that drought can activate otherwise quiescent biosynthetic capacities rather than merely modulating basal metabolic fluxes. Such behavior underscores the limitations of compositional analyses alone and emphasizes the added interpretive value of ratio-based approaches for capturing stress-induced metabolic remodeling. Importantly, this form of qualitative reprogramming, rather than uniform shifts in total GLS content, is consistent with earlier metabolomic and genetic studies demonstrating strong genotype × environment interactions in GLS regulation [34,35,36,37,38]. In this context, the emergence of contrasting response tendencies in the present work, one characterized by enhanced accumulation of indolic and aromatic GLSs (e.g., GBS and GST) and another dominated by selective increases in aliphatic GLSs such as glucoerucin, provides further evidence that drought reshapes GLS profiles through pathway-specific regulation rather than uniform induction or suppression.

Aliphatic GLSs exhibited the greatest heterogeneity in drought responsiveness, particularly among the BR and CCP accessions. In BR3, the near-exclusive induction of gluconapin (GNA) under stress, coupled with strong repression of longer-chain derivatives, suggests a biochemical prioritization of early-chain aliphatic GLS synthesis. This pattern is compatible with constraints related to sulfur availability or biosynthetic cost, as shorter-chain GLSs require fewer elongation and modification steps than their long-chain counterparts [39]. The coexistence of contrasting aliphatic GLS response patterns within the same species complex further underscores the absence of a single optimal biochemical strategy under drought conditions and supports the existence of multiple GLS-based response modes shaped by genetic background [37,40].

Indolic glucosinolates displayed compound-specific regulation rather than uniform induction under drought stress. In several accessions (e.g., BR1, CCP1, BH3), glucobrassicin (GBS) accumulation was accompanied by a concomitant reduction or limited induction of neoglucobrassicin (NGBS), indicating a shift within the indolic GLS pathway rather than a global increase in indolic GLSs. This pattern suggests drought-associated reprogramming of indolic GLS modification steps, likely reflecting altered metabolic flux between core indolic biosynthesis and secondary modification processes. Such differential regulation within the indolic GLS pathway is consistent with previous reports describing the high regulatory plasticity of indolic GLSs under abiotic stress, where they have been implicated in stress-responsive metabolic adjustment and signaling-related processes [10,41,42]. The marked induction of NGBS in CV2, accompanied by suppression of most other GLSs, is particularly notable and points to a highly specialized, accession-specific biochemical response. Aromatic GLSs, represented primarily by gluconasturtiin (GST) and sinalbin (SIB), also displayed pronounced stress responsiveness in specific accessions, with GST showing some of the highest fold increases across the dataset. Given the distinct degradation products and biological activities associated with aromatic GLSs, their selective induction is more likely to reflect accession-specific ecological or physiological specialization than a generalized drought response [43,44].

Based on overall response profiles, the accessions could be broadly categorized into those exhibiting relative biochemical stability (e.g., BH1, BH2) and those showing high metabolic plasticity (e.g., BR3, CCP2, CV1). Importantly, biochemical stability should not be interpreted as an absence of stress responsiveness; rather, it may indicate constitutively optimized GLS configurations requiring minimal adjustment under moderate drought [10]. Conversely, highly plastic profiles reflect a capacity for rapid metabolic reprogramming in response to environmental perturbation [45]. Notably, metabolic plasticity was not necessarily associated with increased GLS diversity. In several accessions, drought triggered a pronounced shift toward one or two dominant compounds rather than diversification [38,46,47], indicating that drought adaptation may favor metabolic streamlining and specialization. Such simplification likely enhances biosynthetic efficiency while maintaining essential defensive or signaling functions. In this context, the coordinated GLS suppression observed in accessions such as BH3 and CCP2 challenges the common assumption that stress tolerance is coupled with GLS induction. Instead, these profiles are consistent with drought response strategies based on metabolic restraint and energy conservation, a mechanism well established in drought physiology but rarely incorporated into GLS-centered models [48,49,50].

From an applied perspective, the strong genotype-dependent divergence in GLS responses has important implications for breeding strategies aimed at improving drought resilience and nutritional quality [8,22]. Biochemical profiling under stress conditions reveals response patterns not captured by control-based screening alone, suggesting that incorporation of stress-responsive metabolite data may enhance the identification of genotypes with balanced adaptive and quality-related traits [12]. However, excessive accumulation of specific GLSs under drought may also raise concerns related to food quality or safety, depending on compound identity and concentration, highlighting the need for careful evaluation of stress-induced metabolic trade-offs [20].

Glucosinolates show strong tissue-specific distribution in Brassicaceae, reflecting developmental regulation and functional specialization. Since leaves were analyzed in the present study, it is relevant that they typically accumulate substantial levels of indolic and short-chain aliphatic GLSs, consistent with roles in defense and stress perception. In contrast, roots often exhibit distinct aliphatic or aromatic GLSs linked to belowground interactions [11], while seeds and young tissues are enriched in GLSs protecting vulnerable developmental stages [51,52]. Beyond defense, leaf GLSs and their hydrolysis products have also been implicated in abiotic stress signaling, redox homeostasis, and hormonal crosstalk [53,54]. Therefore, the drought-driven remodeling of leaf GLS profiles observed here should be interpreted within this spatial context, while recognizing that other organs may follow distinct stress response trajectories.

### 3.2. Correlation Network Shifts Reveal Flux Redistribution Under Drought

The shift in glucosinolate (GLS) correlation patterns between control and drought conditions indicates substantial rewiring of GLS metabolic coordination in Brassica oleracea under water deficit. Under optimal irrigation, negative correlations among major aliphatic GLSs, such as SIN–GRA and GER–GBN, suggest antagonistic accumulation consistent with competitive flux partitioning at constrained metabolic branch points rather than additive biosynthesis, as previously reported in Arabidopsis and Brassica systems [24,34,35,36]. Similarly, the negative GBN–GBS correlation (r = −0.58) and the absence of association between GRA and NGBS support limited coordination between aliphatic and indolic branches under non-stress conditions, consistent with largely independent regulatory modules [29,38].

Under drought conditions, the correlation network reorganized markedly, including inversion of the SIN–GRA relationship (r = +0.57) and emergence of strong positive associations involving SIB, particularly with SIN (r = +0.75) and GER (r = +0.71), indicating increased coordination across GLS classes. Such stress-driven co-regulation has been observed in transcriptomic and metabolic studies of abiotic stress responses [39] and suggests redistribution of metabolic constraints that normally partition GLS biosynthesis. Concurrently, new negative correlations within the aliphatic class (e.g., GER vs. GBAS, GER vs. GAL) point to selective flux redistribution among aliphatic derivatives under drought. Within the indolic class, the negative GBS–NGBS correlation (r = −0.22) further suggests differential regulation of downstream modification steps, consistent with known variability in hydroxylation and methoxylation control in Brassicaceae [36].

Overall, the increased number and strength of correlations under drought supports a shift toward tighter GLS network coupling, consistent with stress-induced canalization and coordinated metabolic adjustment under adverse conditions [41]. From an applied perspective, drought-stable or drought-enhanced associations (e.g., SIN–SIB, GER–SIB) may represent metabolic signatures of responsiveness, whereas stress-specific antagonisms highlight pathway trade-offs relevant for selecting genotypes with balanced metabolic and agronomic performance.

### 3.3. Drought-Driven Transcriptional Reprogramming of GLS Biosynthesis

The drought-responsive transcriptional profiles of glucosinolate (GLS)-related genes revealed a strongly accession-dependent reprogramming of pathway regulation, supporting the view that GLS metabolism is not governed by a single conserved drought-response program but instead emerges from genotype-specific regulatory architectures. The hierarchical clustering pattern, ranging from coordinated co-induction modules (e.g., CCP2/BR1/CV1) to repression-dominant profiles (CCP1) and selective gene-level uncoupling (e.g., BR2, BH1, BR3, CV2), highlights that drought stress can modulate GLS biosynthesis through multiple transcriptional trajectories. Such variability is consistent with the concept that plant secondary metabolism exhibits substantial genotype × environment interaction, and that transcript-level remodeling may partially explain heterogeneous metabolic outputs among Brassica accessions under stress, in agreement with metabolomics-based frameworks for abiotic stress dissection proposed by Obata and Fernie [55].

A major outcome is the differential regulation of MYB transcription factors controlling pathway commitment. The coordinated responsiveness of MYB regulators (notably MYB28/MYB29 for aliphatic GLS and MYB34/MYB122 for indolic GLS) emphasizes their role as upstream integrators of pathway-level transcriptional shifts. This finding aligns with functional studies demonstrating that MYB28 is a key positive regulator of aliphatic GLS biosynthesis in Brassica crops, and that its modulation has been linked to measurable variation in aliphatic GLS accumulation [56]. Importantly, the observed divergence in MYB responsiveness across accessions indicates that MYB-mediated control is environmentally plastic and genetically constrained, suggesting that drought responsiveness may depend not only on the presence of biosynthetic enzymes but also on the capacity to mobilize the regulatory layer coordinating pathway flux. Notably, MYB activation was not consistently accompanied by uniform regulation of downstream structural and tailoring genes (e.g., FMOGS_OX5, CYP81F4, BoGS_OH), reinforcing that GLS control is multi-layered and context-dependent.

Sotelo et al. [57] demonstrated that divergent selection in *Brassica oleracea* reshapes GLS composition and is accompanied by coordinated changes in GLS pathway gene expression, indicating that both upstream regulators and downstream biosynthetic enzymes contribute to genotype-specific GLS outputs. The drought-responsive transcriptional patterns observed here extend this concept by showing that abiotic stress can differentially engage discrete co-expression modules across accessions, resulting in coordinated pathway activation, repression-dominant regulation, or partial uncoupling between regulatory and enzymatic components. This supports a two-layer control model in which (i) genetic background defines the baseline architecture and responsiveness of the GLS network, while (ii) drought acts as an environmental modifier that reshapes the magnitude and direction of transcriptional outputs. Under this framework, drought can be viewed as a selective “regulatory filter” that exposes and amplifies pre-existing genetic variation in transcriptional plasticity, thereby contributing to accession-specific metabolic trajectories.

A further key feature is the strong accession-dependent regulation of downstream tailoring genes such as FMOGS_OX5 and CYP81F4, indicating that drought does not solely affect pathway commitment via MYBs but can also reconfigure derivative-specific modification steps. This is particularly relevant because 2-oxoglutarate-dependent dioxygenases are established determinants of GLS structural outcomes; for example, Kakizaki et al. [58] functionally linked such an enzyme to glucoraphasatin biosynthesis in radish. Thus, the variable FMOGS_OX5 responsiveness observed here suggests that drought may preferentially impact enzymatic diversification nodes, promoting qualitative shifts in GLS composition rather than uniform changes in total GLS output. The recurrent uncoupling between MYB regulators and downstream tailoring genes further challenges a purely MYB-driven linear model of GLS transcriptional control under drought and suggests that modification steps may be shaped by additional regulatory layers, including alternative transcriptional programs, post-transcriptional constraints, or metabolic feedback, thereby limiting the predictive value of MYB induction alone.

The drought-responsive profiles also implicate CYP-dependent steps as potential drivers of accession-specific GLS remodeling. Cytochrome P450 enzymes catalyze committed reactions that constrain pathway entry and diversification [59], and the strong inter-accession variability observed here suggests that drought may redistribute regulatory control across such biosynthetic nodes. Although only a subset of GLS genes was profiled, these patterns align with transcriptomic evidence indicating that GLS networks are highly plastic across Brassica developmental contexts [60], reinforcing that GLS regulation is not fixed and can be reprogrammed at multiple levels under stress.

Overall, the transcriptional patterns converge on three non-exclusive modes of drought-responsive GLS regulation: (i) coordinated co-induction of MYB regulators and core biosynthetic genes, (ii) repression-dominant attenuation of GLS transcription, and (iii) selective uncoupling between upstream regulators and downstream tailoring steps. These contrasting configurations indicate that drought can reprogram GLS metabolism through accession-specific regulatory architectures. Importantly, transcript shifts cannot be assumed to translate proportionally into GLS accumulation because turnover, transport, compartmentalization, and hydrolysis can substantially reshape metabolite outputs.

### 3.4. Transcript–Metabolite Coupling Highlights Drought-Responsive GLS Control Points

To connect transcriptional variation with metabolic outputs, gene–metabolite correlation analysis was used to identify compound-resolved associations consistent with flux control points within the GLS network under drought. Strong positive associations between MYB28, MYB122, and sinigrin (SIN) support coordinated regulation linking these transcription factors to aliphatic GLS accumulation, in agreement with the established regulatory role of MYB28 in Brassica [56] and with prior pathway-level evidence [28]. Importantly, these correlations should be interpreted as statistical coupling rather than direct proof of causality, since coordinated transcript–metabolite behavior can also arise from shared upstream signaling inputs or broader co-regulation of pathway activity.

In contrast, MYB29 displayed divergent correlation signatures, with a moderate negative association with SIN (r = −0.409) but a strong positive association with glucoraphanin (GRA) (r = 0.883), suggesting that MYB29 may be linked to branch-level redistribution among aliphatic GLS derivatives under drought, consistent with context-dependent MYB29 functions reported in Brassica [56,57,58,59,60]. MYB34 showed weaker associations with aliphatic GLSs and a notably weak correlation with GRA (r = −0.276), supporting a more specialized regulatory involvement rather than uniform control across the aliphatic branch. At the enzymatic level, ST5a and ST5b were positively correlated with SIN (r = 0.782 and 0.777), reinforcing the potential contribution of terminal sulfation steps to shaping aliphatic GLS output [61]. Notably, the strong negative association between ST5a and GRA (r = −0.841) indicates that late pathway reactions may impose derivative-specific constraints, favoring selective routing between aliphatic products rather than constitutive end-point assembly.

Additional evidence for compound-specific control was provided by the strong negative correlations between gluconapin (GNA) and the tailoring-related genes CYP81F4 and BoGS_OH (r = −0.791 and −0.610, respectively), consistent with trade-offs in aliphatic GLS allocation potentially mediated through modification-related processes [62]. Although GNA has been associated with antioxidant-related functions, caution is required when inferring physiological roles directly from correlation patterns, since GLS abundance is strongly influenced by turnover, transport, compartmentalization, and hydrolysis in addition to biosynthetic flux [63,64]. From an applied perspective, identifying regulatory and enzymatic nodes statistically associated with nutritionally relevant GLSs such as SIN and GRA remains valuable, given their recognized health-promoting properties and relevance for crop improvement [10].

Finally, drought-driven GLS remodeling is frequently shaped by hormonal and resource allocation shifts that can modify transcriptional hierarchies and metabolic flux partitioning [65]. Stress-associated signaling pathways involving ABA, JA, and ethylene have been implicated in modulating GLS regulators, including MYB transcription factors, thereby providing a plausible upstream context for the coordinated transcription–metabolite relationships observed here [65,66,67]. However, because hormone dynamics were not quantified in this study, these signaling pathways should be regarded as biologically plausible contributors rather than confirmed drivers of the observed coupling patterns.

### 3.5. Protein Interaction Networks and Regulatory Integration in GLS Pathway Control

Transitioning from gene expression to a broader perspective, the protein–protein interaction network complements this understanding by revealing the intricate molecular dialogs that underpin pathway regulation. TFs, as central players in gene expression, often occupy pivotal positions in these interaction networks, further elucidating their roles in shaping the glucosinolate biosynthesis pathway [68,69]. In plant genetics, gene expression is commonly understood to be regulated through the interaction of promoters with specific transcription factors, facilitated by direct binding to cis-acting regulatory elements (CAEs) [70]. The clustering of proteins suggests a coordinated response to diverse environmental cues, delicately adjusting the biosynthesis of GLS compounds. Such regulatory precision is crucial for bolstering plant defense mechanisms against herbivores and pathogens, while also influencing the flavor profile and nutritional quality of cruciferous vegetables [71]. However, an experimental verification of these regulatory interactions (e.g., via reporter assays, ChIP-qPCR or mutant analysis) would be necessary to confirm their functional roles in a future study.

Genotype-dependent variation in GLS accumulation and correlation structure supports a highly plastic metabolic architecture under drought, reflecting accession-specific strategies rather than uniform pathway regulation. The combined metabolite- and transcript-level signatures indicate that drought reshapes GLS outputs through coordinated shifts in regulatory modules and downstream tailoring capacity, with potential implications for both stress adaptation and quality-related traits. These patterns provide a mechanistic basis for identifying responsive genotypes and prioritizing candidate regulatory nodes for breeding-oriented selection. Future work integrating functional validation with broader omics approaches will be essential to confirm causality and translate these associations into robust markers for drought resilience and targeted GLS optimization in cruciferous crops.

## 4. Materials and Methods

### 4.1. Plant Materials and Growth Conditions

The study included landraces (LRs) of *Brassica oleracea* and composite cross populations (CCP) developed within the framework of the EU H2020 BRESOV project (Table 1). The plant materials investigated comprised landraces of cauliflower (*B. oleracea* L. var. *botrytis*), broccoli (*B. oleracea* L. var. *italica*), and kale (*B. oleracea* L. var. *acephala*), along with CCP F_1_ and F_2_ generations.

A split-plot experimental design was employed, incorporating two factors: the irrigation regime (IR) and genotype (GE). Each treatment was replicated three times, with ten plants per elementary plot. The study included twelve genotypes: three kale varieties (BH1, BH2, BH3), three broccoli accessions (BR1 to BR3), three cauliflower accessions (CV1 to CV3), and three composite cross populations (CCP1 to CCP3). All plant materials were sourced from the *Brassica* collection of the Department of Agriculture, Food, and Environment (Di3A) at the University of Catania (UNICT). A sufficient number of seeds from each accession were sown in cellular trays using an organic substrate (Terri Bio, “Agro-Chimica S.p.,” Bolzano, Italy) and maintained under cold greenhouse conditions at the experimental farm of the University of Catania (Di3A) (Southern Italy, 37°31′10″ N, 15°04′18″ E). During September 2021, the experimental site experienced an average temperature of approximately 24 °C, with daily highs reaching 34 °C and lows around 16 °C. The relative humidity averaged 71%, and total precipitation for the month amounted to about 49 mm, distributed over approximately eight rainy days. The site received around 12 h and 23 min of daylight per day, with approximately 11 h of sunshine. After one month, seedlings were transplanted into 0.3 L pots filled with the same substrate used for sowing. Four weeks after transplantation, plants of each accession were separated into two treatment groups: (i) an irrigated control groups (IRR), where 10 plants of each accession were watered to field capacity, and (ii) a non-irrigated group (NIR), where 10 plants of each accession were subjected to a progressive water deficit regime by reducing irrigation from 100% field capacity to complete water withholding (0% field capacity) to induce drought stress. Once 0% was reached, plants were maintained under these conditions for 14 consecutive days. This duration was chosen to induce a stable physiological and molecular response to water deficit, while avoiding irreversible damage or plant death [10]. Following two weeks of water deficit regime (Figure S1), plants were harvested for biochemical assessments. Leaf samples were carefully washed, dried and stored at −80 °C for one week before being freeze-dried for further biochemical analysis.

### 4.2. Analytical Procedure for Glucosinolate Extraction and Quantification by HPLC

Glucosinolate (GLS) extraction was performed following the International Standard Method ISO 9167-1 (1992) [72], with modifications and formal adoption by the European Commission (1990). Glucosinolate was extracted from 200 mg of freeze-dried leaves, randomly collected from each accession in two water treatments (three replicates × 12 genotypes × 2 treatments). For each sample, extraction was performed in 5 mL of 70% methanol at 70 °C for 10 min to inactivate myrosinase. The extract was then centrifuged at 12,000 rpm for 20 min at 4 °C, and the resulting supernatant was collected. To obtain desulfoglucosinolates, 2 mL of the supernatant was loaded onto a DEAE-Sephadex A-25 resin column (0.5 mL) and hydrolyzed with sulfatase. The content and profile of glucosinolates (GLSs) were determined using the High-Performance Liquid Chromatography HPLC Agilent 1200 series system (Agilent, Santa Clara, CA, USA) equipped with a diode array detector (DAD). Desulpho-glucosinolates were separated using this technique. A stock standard solution was prepared by dissolving 0.2 M of each of the ten intact glucosinolate standards in 2 mL of Milli-Q water. This stock solution was then serially diluted to obtain calibration standard solutions at concentrations of 0.1, 0.2, 0.4, and 1.0 µmol·mL^−1^. All standard solutions were stored at 4 °C until analysis. Desulpho-glucosinolates were extracted from the samples and injected into an HPLC system equipped with a Diode Array Detector (DAD) and a Kinetech C18 column (250 × 4.6 mm, particle size 5 µm). The mobile phase consisted of two solvents: solvent A (ultrapure water) and solvent B (acetonitrile: water, 20:80, *v*/*v*). The flow rate was set at 1.1 mL·min^−1^, and the injection volume was 20 µL. A binary gradient program was used as follows: 100% A for 5 min, followed by a linear increase to 70% A and 30% B over 12 min, and then 30% A and 70% B for 3 min. The total run time was 40 min. The chromatograms were recorded at a wavelength of 229 nm. The quantification of each glucosinolate was performed based on the calibration curves derived from the external standards (Table S3). All the standards were purchased from ChromaDex (Santa Ana, CA, USA). Retention times (RT) and UV spectra were used to identify and quantify the compounds. All experiments were conducted in triplicate.

### 4.3. RNA Extraction and cDNA Synthesis

Total RNA was isolated from leaf tissues of each accession in two water conditions (three replicates × 12 genotypes × 2 water treatments), using the RNeasy Plant Mini Kit (Qiagen, Hilden, Germany) with on-column DNase I treatment to eliminate genomic DNA contamination. RNA concentration and purity were determined with a NanoDrop 1000 spectrophotometer (Thermo Fisher Scientific, Waltham, MA, USA), while RNA integrity was assessed following the protocol of Masek et al. [73]. Briefly, 2 µg of total RNA was denatured at 65 °C for 5 min in a mixture of 50% (*v*/*v*) formamide, 1× TAE buffer, 5% (*v*/*v*) glycerol, and 0.025% (*w*/*v*) bromophenol blue, immediately chilled on ice, and separated on a 1% (*w*/*v*) agarose gel. For cDNA synthesis, 1.5 µg of total RNA was reverse-transcribed using the RevertAid H Minus First Strand cDNA Synthesis Kit (Thermo Fisher Scientific, formerly Fermentas, Bermen, Germany) with an oligo(dT)18 primer, following the manufacturer’s instructions.

### 4.4. Quantitative Real-Time PCR (qRT-PCR)

Quantitative real-time PCR was performed on a Rotor-Gene Q cycler (Qiagen, Hilden, Germany) using QuantiSpeed SYBR Green Mix (Qiagen). Each 20 µL reaction contained 10 µL of 2× SYBR Green mix, 1 µL of forward and reverse primers (10 µM; Table 2), 1 µL of cDNA template (equivalent to 100 ng RNA), and 7 µL of nuclease-free water. The thermal cycling program consisted of initial denaturation at 95 °C for 10 min, followed by 40 cycles of 95 °C for 15 s, 59 °C for 15 s, and 72 °C for 10 s. A final extension at 72 °C for 10 min and melt-curve analysis were included to verify amplification specificity. All reactions were performed in three technical replicates, and reference genes were used for normalization in accordance with MIQE guidelines.

### 4.5. Data Analysis

For the visualization of glucosinolate response patterns, a heat map was generated to represent relative changes in glucosinolate accumulation across accessions and experimental conditions using R Studio (R v4.4.2) and the pheatmap package. Heat maps were constructed using log_2_-transformed fold-change values calculated as log_2_(stress/control) from absolute glucosinolate concentrations, enabling symmetric visualization of induction and repression. Z-score normalization was applied by glucosinolate to facilitate comparison of response patterns across accessions. Hierarchical clustering was performed using Euclidean distance and Ward’s linkage method and was applied as a descriptive approach to group accessions according to similarity in glucosinolate response profiles and to identify major response clusters under drought stress.

The normalized gene expression levels were calculated using the fold induction method, as described by Livak and Schmittgen (2001) [74]. The formula applied was

Fold induction = 2^−ΔΔCt^

where ΔΔCT is calculated as follows:

ΔΔCT = (CT _Gene stressed_ − CT _Actin stressed_) − (CT _Gene control_ − CT _Actin control_)

In this calculation, Ct Gene denotes the cycle threshold of the target gene, while Ct Actin corresponds to the cycle threshold of the housekeeping gene used for normalization. This normalization corrects for sample-to-sample variation in RNA quantity and quality, enabling accurate assessment of relative expression changes under stress conditions. Data acquisition and analysis were performed with Q-Rex software (Version Number version 2.0) (Qiagen, Hilden, Germany) to ensure standardized quantification.

Pearson’s correlation coefficients (r) were calculated to assess linear relationships among the studied variables using the “cor” function from the stats R Studio (version 4.4.2). Correlation matrices were visualized using heatmaps (“corrplot package”) (version 0.95). Correlation strength was interpreted according to the magnitude of correlation coefficient (r), classified as weak (|r| < 0.2), moderate (0.2 ≤ |r| < 0.5), and strong (|r| ≥ 0.6) [75]. For the visualization of gene expression data, a heatmap was generated to represent the relative expression levels across different experimental conditions using R Studio software (version 4.4.2), employing the “pheatmap” package (version 1.0.13). The clustering method used was the complete linkage algorithm with Euclidean distance, allowing for the grouping of genes with similar expression profiles and the identification of distinct sample clusters.

Protein interactions were analyzed using the STRING v11.0 database (https://string-db.org), a widely used platform for exploring known and predicted protein–protein interactions. The analysis focused on proteins with the highest interaction scores, which were identified through a similarity search against the Arabidopsis proteome in the STRING v11.0 database. These proteins, which exhibited significant interaction evidence, were selected for further analysis to elucidate potential functional relationships within the biological network. This analysis provides putative functional associations rather than experimental validation of gene function.

## 5. Conclusions

This study reveals that *Brassica oleracea* exhibits pronounced genotype-dependent plasticity in glucosinolate (GLS) biosynthesis under drought stress. Differential accumulation patterns, particularly the upregulation of aliphatic and indolic GLSs in accessions such as BR2 and BR3, underscore the role of secondary metabolism in abiotic stress adaptation. Conversely, genotypes like CCP2 and BH3, which displayed GLS downregulation, may employ alternative energy-conserving responses. The reprogramming of GLS pathways, evidenced by shifts in compound levels and inter-correlations, suggests tight regulation by stress-responsive transcriptional networks. Notably, the induction of glucobrassicin (GBS) highlights the functional significance of indolic GLSs as potential markers of regulatory flexibility. The involvement of key regulatory loci such as MYB28, MYB29, MYB34, and ST5a further supports their central role in modulating stress-induced metabolic responses. These findings provide a robust framework for incorporating GLS profiling into precision breeding strategies aimed at improving drought resilience and phytochemical composition in *B. oleracea*. Future research should integrate multi-omics approaches to dissect the regulatory networks underlying this metabolic adaptability.

## Figures and Tables

**Table 1:** List of *Brassica* accessions.

Crop Type	Commun Name	Id Bresov	UNICT Code	Morphotype	Crop Code
Landraces (LRs)	Kale	B1800061	UNICT364	*B. oleracea* var. *acephala*	BH1
		B1800077	UNICT4538	*B. oleracea* var. *acephala*	BH2
		B1800071	UNICT4591	*B. oleracea* var. *acephala*	BH3
	Broccoli	B1800082	UNICT3122	*B oleracea* var. *italica*	BR1
		B1800083	UNICT613	*B oleracea* var. *italica*	BR2
		B1800074	UNICT656	*B oleracea* var. *italica*	BR3
	Cauliflower	B1800985	UNICT5098	*B.oleracea botrytis*	CV 1
		B1800987	UNICT5100	*B.oleracea botrytis*	CV 2
		B1800988	UNICT5101	*B.oleracea botrytis*	CV 3
Breeding lines	composite cross populations (CCP)	B1800951	UNICT5041	*B oleracea* var. *incrocio*	CCP1
		B1800954	UNICT5044	*B oleracea* var. *incrocio*	CCP2
		B1800959	UNICT5049	*B oleracea* var. *incrocio*	CCP3

**Table 2:** Primer sequences and efficiency of GLS biosynthesis-related genes used in relative expression analysis via qPCR.

Name	Accession ID Number	cDNA Size (bp)	Forward Primer Sequence	Reverse Primer Sequence	Product Size (bp)	Primer Efficiency Values (%)
	**Transcription Factor-Related Genes**					
*MYB28*	Bol007795	558	CCACACCAGTTCAGAGAGGT	GGGAAATGGATCGAAGTCAGC	221	98
*MYB29*	Bol008849	513	CGCCCAAGACTTCTGAGTT	TGATATTGCCCATGGAAGCTG	234	95
*MYB34*	Bol017062	951	AAGGTGGATGGCGTACTCTC	TGTGAGTGGTTGGATCGACA	279	98
*MYB122*	Bol026204	981	GACCATTCCGAGACATTGCC	GCATCGTGGATCATGTGGAG	284	94
	**Aliphatic Biosynthesis-Related Genes**					
*ST5B*	Bol026202	1035	AAGCCTTGACTTTCGCCATC	ACTTCACAACTGAGTCCGGT	204	100
*FMOGS-OX5*	Bol031350	1380	ATGGCACCCTCTTGCAGTCC	AGTCGTAGACGCTAGAGTGG	226	99
*GSL-OH*	Bol033373	243	GATTGTGCAAAAGGCTTGT	AGAGCATTAGGATTAGGAGGA	188	96
	**Indolic Biosynthesis-Related Genes**					
*ST5A*	Bol026200	1017	GTCCGGTTGCAAGATGGTTT	CCTCTCCGGGTTCTCTTTGT	214	100
*CYP81F1*	Bol028914	1497	CTTTCCAACTGACGGCCAAA	CGTTAGGTCCGAGAAAAGCG	257	99

## Data Availability

The original contributions presented in this study are included in the article and Supplementary Materials. Further inquiries can be directed to the corresponding author.

## Supplementary Materials

The following supporting information can be downloaded at https://www.mdpi.com/article/10.3390/ijms27031598/s1.
