# Supplementary material for: Integrating Metabolic and Gene Expression Profiling of Glucosinolate Biosynthesis Under Drought Stress in Brassica oleracea
Source: Int J Mol Sci. 2026 Feb 6;27(3):1598. doi: 10.3390/ijms27031598 (PMC12898713; doi:10.3390/ijms27031598)
Supplement: Supplementary file 1 [file ijms-27-01598-s001.zip › Figure S1.pdf]

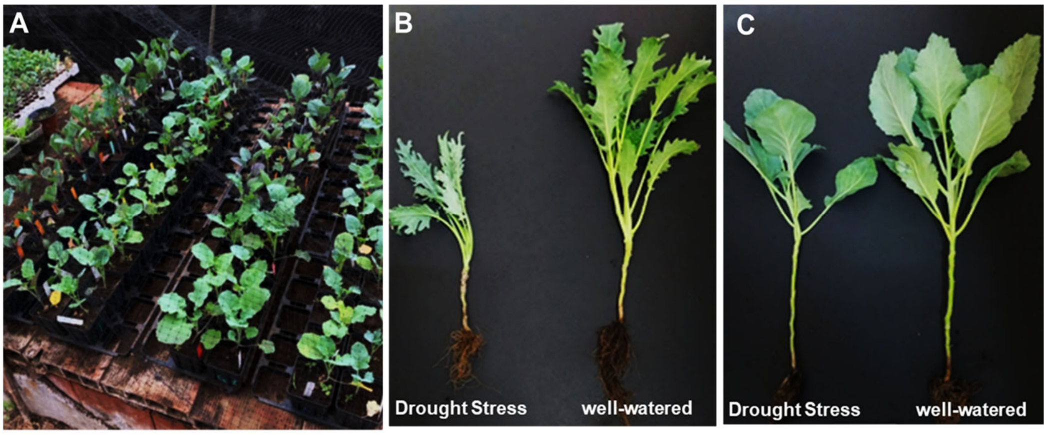

Figure S1: (A) A greenhouse pot experiment conducted under controlled drought stress conditions, (**B**, **C)** representative phenotypic differences among the studied *Brassica* accessions under control and drought stress treatments.
